# Imaging in Vascular Access

**DOI:** 10.1007/s13239-017-0317-y

**Published:** 2017-07-13

**Authors:** Eoin A. Murphy, Rose A. Ross, Robert G. Jones, Stephen J. Gandy, Nicolas Aristokleous, Marco Salsano, Jonathan R. Weir-McCall, Shona Matthew, John Graeme Houston

**Affiliations:** 1 Metabolic and Clinical Medicine, College of Medicine, Ninewells Hospital, University of Dundee, Dundee, DD1 9SY UK; 2 NHS Tayside Vascular Department, Ninewells Hospital, Dundee, DD1 9SY UK; 3 Department of Interventional Radiology, Queen Elizabeth Hospital Birmingham, University Hospital Birmingham, Birmingham, B15 2WB UK; 4 NHS Tayside Clinical Radiology, Ninewells Hospital, Dundee, DD1 9SY UK; 5 NHS Tayside Medical Physics, Ninewells Hospital, Dundee, DD1 9SY UK

**Keywords:** Arteriovenous fistula, Medical imaging, Vascular access, Ultrasound, Digital subtraction angiography, Magnetic resonance imaging, Computed tomography

## Abstract

This review examines four imaging modalities; ultrasound (US), digital subtraction angiography (DSA), magnetic resonance imaging (MRI) and computed tomography (CT), that have common or potential applications in vascular access (VA). The four modalities are reviewed under their primary uses, techniques, advantages and disadvantages, and future directions that are specific to VA. Currently, US is the most commonly used modality in VA because it is cheaper (relative to other modalities), accessible, non-ionising, and does not require the use of contrast agents. DSA is predominantly only performed when an intervention is indicated. MRI is limited by its cost and the time required for image acquisition that mainly confines it to the realm of research where high resolution is required. CT’s short acquisition times and high resolution make it useful as a problem-solving tool in complex cases, although accessibility can be an issue. All four imaging modalities have advantages and disadvantages that limit their use in this particular patient cohort. Current imaging in VA comprises an integrated approach with each modality providing particular uses dependent on their capabilities. MRI and CT, which currently have limited use, may have increasingly important future roles in complex cases where detailed analysis is required.

## Introduction

Haemodialysis is the most common treatment for end stage renal disease (ESRD). To achieve successful haemodialysis, a functional vascular access (VA) capable of managing 300–400 mL extraction of blood per minute is essential.^10^ There are three main types of VA, autogenous arteriovenous fistula (AVFs), prosthetic AV grafts (AVGs) and central venous catheters (CVCs).

An AVF is considered the preferred VA because of its association with prolonged survival, fewer infections, lower hospitalisation, and reduced costs in comparison with an AVG or CVC.^56^, ^61^, ^93^ An AVF is a surgically created anastomosis joining a peripheral artery and vein. The introduction of arterial blood flow, and its associated increased blood pressure, into a vein should induce vein expansion and cause its walls to remodel. This expansion and remodelling, known as maturation, allows regular cannulation and is critical for successful long-term haemodialysis; however, some AVFs fail to ever mature.

Where the patient’s vessels are deemed unsuitable for the formation of an AVF, an AVG can be used. AVGs involve connecting an artery to a vein *via* a tube commonly made from expanded polytetrafluoroethylene (ePTFE), although other synthetic and biological grafts are available.^23^ The risk of infection for AVGs is significantly higher than AVFs, 16 and also, AVGs require more interventions than AVFs post-implantation to maintain long-term graft patency.^5^

AVFs and AVGs are unusable for haemodialysis if they are incapable of the required extraction flowrates. A stenosis, which can result in thrombosis, is the most common late complication.^16^, ^80^ Stenoses are most commonly caused by the development of myointimal hyperplasia at the venous anastomosis site and require intervention to maintain patency.^16^, ^80^ Other problems associated with AVFs and AVGs are steal syndrome, pseudoaneurysms and aneurysms, extrinsic abnormalities, compressing haematoma/seroma, congestive heart failure, and infection, although these may not necessarily inhibit use for haemodialysis.^16^, ^80^

CVCs, on the other hand, provide short—medium term access in urgent or emergent situations, but are more prone to high rates of failure and infection, and are associated with higher mortality.^49^ CVCs are plastic tubes with two lumens, one for blood extraction and one for blood return, that are inserted into a central vein commonly terminating at or within the right atrium. Inlet and outlet ports are provided for dialysis that protrude from the incision site. For patients who switch from CVC to an AV access there is an approximate 50% reduction in mortality^3^; however, AVF survival is reduced for patients who previously had a CVC in comparison with those who did not.^67^ One of the reasons for this is because CVCs can cause local intimal injury, with endothelial denudation and thrombus, within the central veins that can lead to central vein stenosis.^27^

The heterogeneous nature of ESRD and the natural history of all three VA types is further complicated by the significant comorbidities present in this cohort of patients, such as diabetes and peripheral vascular disease. Given the haemodynamic nature of many of the issues in VA, and that the local haemodynamics can change throughout their lifetime, medical imaging has a critical role. Some centres use medical imaging to monitor patients’ vasculature pre- and post-operation for rapid diagnosis and management of VA related complications. Also, imaging has a research role in elucidating the underlying issues that may result in VA failure, the outcome of which could be surgical or design recommendations that contribute to lower failure rates. Up until now, despite extensive research, the ideal conduit remains the holy grail of vascular access.

VA sites, in particular AVFs, are commonly imaged using ultrasound (US), but other modalities can also be used: digital subtraction angiography (DSA), magnetic resonance imaging (MRI), and computed tomography (CT). The use of all four imaging modalities in VA is reviewed here with a discussion of their individual advantages and disadvantages, and possible future directions.

## US

Since its inception US has provided important diagnostic information in the clinical context and particularly in the 1970s technological advances led to widespread use of US imaging in medical diagnosis. Real time b-mode (2-dimensional (2-D)) imaging replacing the primitive static A-mode (1-D) and b-mode images, allowing cross sectional images without the use of ionising radiation. This development had significant implication most notably in obstetrics where application of this technique progressed from simple measurements of anatomical dimensions, such as femur length and biparietal diameter, to detailed screening for foetal abnormalities.

Colour Doppler provides non-invasive assessment of general vasculature of the abdomen and peripheral circulation without the use of ionising radiation or contrast especially in those patients whose kidney function is compromised. In many areas US is the initial imaging modality of choice in the investigation of arterial disease. Colour Doppler US has a proven ability as a diagnostic imaging procedure more recently in VA.^58^ Tordoir first recommended US in the assessment of fistula,^89^ and today it has two pivotal roles: (a) preoperative assessment of both arteries and veins, (b) post-operative assessment of fistula maturation and possible complications.

### Primary Uses in VA

US is an integral part of the haemodialysis service and can be routinely used both in the preoperative assessment and assessment where dialysis dysfunction has been identified, and in some centres is part of an aggressive surveillance and intervention regime to maintain patency. The challenge however is to predict whether an AVF will mature to provide an efficient and reliable haemodialysis access. AVFs fail most commonly secondary to stenosis. Guidelines recommend the use of surveillance tools in an attempt to identify fistulas at risk and US fulfils the recommended criteria recognised in mass screening.^102^

#### Preoperative

Guidelines recommend US in the assessment of both the inflow (artery) and the outflow (vein) of a AVF.^88^, ^94^ High frequency transducers (Tx) (14 MHz) allow for detailed morphological and haemodynamic information on both the feeding artery and draining vein to allow the selection of the most appropriate site for the fistula. However, the pulse repetition frequency is a recognised limitation of high frequency Tx and as such, occasionally it may be necessary to resort to a lower frequency Tx (10 MHz) to quantify the high velocities encountered in a stenosed fistula.

##### Arteries

B-mode imaging can assess calibre, uniformity, diameter and the presence of calcium. These are important since arterial diameter is related to fistula outcome^69^, ^76^, ^90^ and calcified vessels can inhibit maturation of the fistula. Colour Doppler US can identify phasicity of the vessel and the presence of proximal or distal disease.^90^ The presence of arterial disease can also be the cause of ischaemic fingers or hand once the fistula is routinely used in haemodialysis which is commonly known as “steal syndrome”.

##### Veins

B-mode imaging is also used to assess veins and can identify thickened valve sites, presence of branches and small non-occlusive thrombus not readily palpable. It can identify veins not readily palpable on clinical examination thus increasing the number of fistula that can be created. Venous cut off diameters range from 1.6 to 2.6 mm.^9^, ^52^, ^69^, ^76^, ^103^ Colour Doppler US can determine patency and identify central venous obstruction by direct visualisation or indirectly through interrogation of the Spectral Doppler waveform where a lack of phasicity in the peripheral vessels can indicate a central stenosis. This assessment must not be taken in isolation. For example, any history of previous tunnelled lines should pre-empt a formal assessment of the central venous system.

It must be noted, paradoxically, that this preoperative assessment may increase the number of forearm and upper arm fistulas created, by identifying suitable vessels at more sites than were apparent clinically^4^, ^14^, ^69^, ^76^; however, it has also been argued it offers no added information.^100^ Despite this, vascular mapping is still an integral part of the preoperative assessment by complementing the physical evaluation in the identification of possible access sites. It still remains that it can increase the number of autologous fistulas and offers extended renal replacement therapy.

#### Post-operative

**Figure 1:**
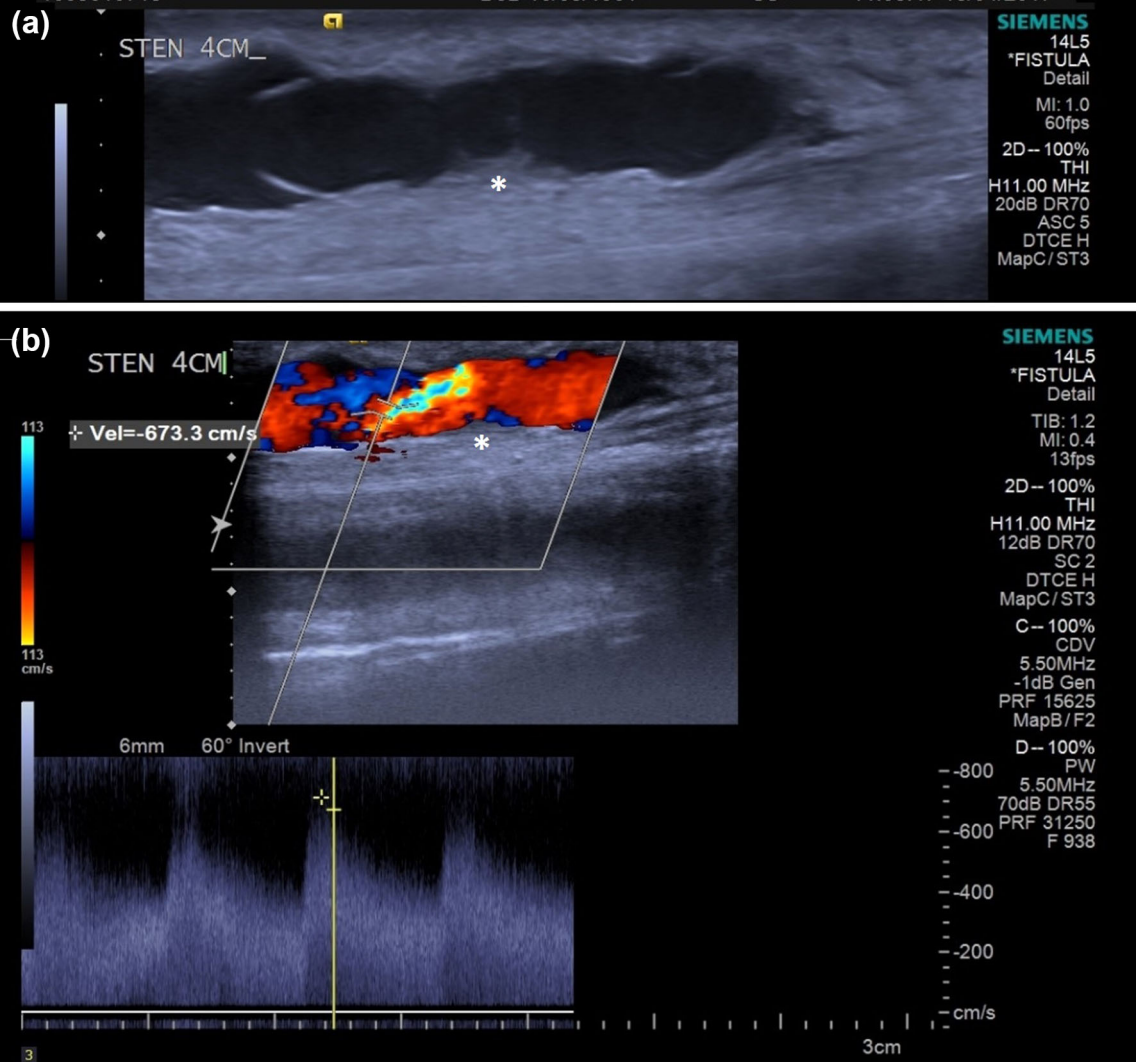
(a) US b-mode image of the vein-side of an AVF with a stenosis (marked with an asterisk). Also visible are the leaflets of a venous valve fixed in position. (b) US b-mode image of the same area superimposed with the colour Doppler data, and with PSV recorded at 6.733 m/s at the location within the crosshairs (stenosis again marked with an asterisk).

Despite the recommendation of US as a surveillance assessment, the National Kidney Foundation’s Kidney Disease Outcomes Quality Initiative (KDOQI) remains committed to clinical evaluation as a determinant of maturation.^72^ The predictive value of US, as with any other imaging modality, is the definition of a clinically significant stenosis. Detailed b-mode assessment can identify intimal hyperplasia associated with stenosis in the AVF.^101^ Additionally, Spectral Doppler analysis can determine the degree of stenosis. A peak systolic velocity (PSV) >3.5 m/s or a PSV at the site of a stenosis three times the PSV before the stenosis are consistent with a significant stenosis.^33^, ^89^ An example showing how the PSV is measured is shown in Fig. 1b where a PSV of 6.733 m/s is measured just downstream of a stenosis site which is more clearly visible in Fig. 1a. Spectral Doppler analysis of the flow central to the stenosis can determine the haemodynamic impact and therefore, the risk of failure. Increased resistance of the feeding artery is a late indirect marker of a dysfunctional fistula, with increasing resistance in the vessel associated with significant stenosis. As with the preoperative assessment it is essential that the assessment is not taken in isolation.

Volume flow is another widely recognised measurement tool for VA post-operative assessment, specifically in the vein segment of the AVF,^12^, ^68^, ^101^ however, this measurement is calculated by the US machine and is based on either the time average velocity (TAVx) or the time average mean velocity (TAMx). Both TAVx and TAMx are calculated by the US machine software, and TAMx particularly is dependent on operator experience. These velocity measurements can lead to significant variations in the measured volume flow. Publications vary in which measurement they use,^12^, ^105^ and therefore, although it can give an indicator of the volume flow, it cannot be considered an accurate measurement.

US in the post-operative period can also provide valuable information in regards to “steal phenomenon” by determining the presence of low or high flow steal. Careful evaluation of the run off vessels and the assessment of the fistula for presence of stenosis can determine the most appropriate intervention to maintain dialysis adequacy. Detailed knowledge of the anatomy of the stenosis acquired using US may also influence the access site for intervention.^22^ A clinical evaluation, together with a brief history of any dialysis issues, is invaluable in the support of any ultrasound findings. A limitation of US is inaccessibility of the central veins and a history of increasing venous pressure or quite simply increased swelling in the arm should necessitate a formal assessment of the central veins.

Outside of assessment of an AVF, US can also be used in guided cannulation of “difficult-to-cannulate” VA. This is becoming increasingly common globally with increasing dialysis patient age and obesity seen as reasons for US guided cannulation^41^, ^98^; although, Harwood *et al*. noted that some nurses remain in a state of ‘perpetual novice’ resulting in negative patient experience (bruising and pain).^36^ This again reflects the importance of sufficient operator experience and skill when using US.

### Advantages/Disadvantages

Colour Doppler US offers an inexpensive non-invasive assessment of the AVF. It is readily available and provides a dynamic assessment of the fistula. Interestingly, in the example shown in Fig. 1 there is a valve located downstream of the stenosis (clearly visible in Fig. 1a) which had become fixed in the position shown. This caused recirculation of the blood on the underside of the valve leaflets, which is visible in Fig. 1b, and could eventually lead to thrombosis and failure of the AVF. Critically, this would not be apparent in a fistulogram. US reduces exposure to ionising radiation and contrast agents, and it can be used as an independent imaging modality as US guided imaging for fistuloplasty.^30^, ^95^ The use of US is not the without its limitations principally in that it is operator dependent, and relies on experienced staff to both perform and interpret the information.^15^, ^101^ US has a limited field of view (FOV) which may be addressed by new advances such as extended FOV. US is a 2-D assessment of a 3-D vessel/organ which increases its reliance on operator experience as some detail of the target area could be missed. 3-D imaging may reduce the impact of this limitation. A further limitation of US is its inability to image the central venous system. The sternum, clavicle and 1st and 2nd ribs limit access to the central veins and therefore the diagnostic potential. As previously discussed it is essential that the assessment of the fistula is not reported in isolation both in the preoperative and post-operative phase.

### Future Directions

US has a proven ability as both a diagnostic and surveillance tool. Preoperative mapping can identify suitable vessels to be used in the formation of a fistula; however, there is however a lack of evidence that any assessment can predict those fistula that will fail. Despite the recommended limit on size of vein or artery,^62^ there is still no clear evidence in support of its predictive nature.^59^ The vascular endothelium provides both a structural and functional role within the body. Endothelial dysfunction is thought to be the first step in atherosclerosis^2^, ^32^, ^64^ and could be an issue in AVF maturation. Endothelial reactivity is a measure of the ability of a vessel to vasodilate in response to a stimulus that promotes nitric oxide formation, such as wall shear stress (WSS). Assessment of endothelial reactivity could be obtained using US. Arterial stiffness is known to be of importance in relation to future cardiovascular morbidity and mortality. Velocity Vector Imaging (VVI) is a novel US analysis simultaneously assessing longitudinal and radial tissue motion. It can be measured in the common carotid artery^83^ and is associated with plaque burden.^84^ This may provide a novel technique in the assessment of AVF maturation. Brachial artery elasticity has been shown to differ between patients with chronic kidney disease (CKD) and healthy volunteers,^77^ and variation within the CKD population may be predictive of maturation of AVF. New technological advances in US have improved the detail and sensitivity, but have no major impact on final diagnosis.

#### Extended FOV

A limitation of US is the limited FOV requiring multiple images to determine anatomical relationships. Extended FOV facilitates panoramic images over several centimetres with no loss of resolution. This allows correlation with anatomical landmarks supporting planned access for intervention.^45^

#### Harmonic Imaging

Harmonic imaging utilises both the fundamental (original) frequency and the harmonic frequency which occurs at twice the fundamental frequency. The harmonic signals are as a result of interaction with body tissues or contrast agents. The harmonic signal has a narrower beam and lower side lobes resulting in improved grey scale contrast resolution.^97^ Harmonic frequencies are, however, most effective in the mid-field where the distortion of the signal is most apparent, and it is for this reason that its impact in the imaging of the fistula is limited given the very superficial nature of the vessels under interrogation.

#### Compound Imaging

Traditionally the US image is represented in a single view 90° to the Tx. Compound imaging uses beam steering and sends the signal from multiple angles which are all received and translated into a single image, resulting in increased resolution. Again, given the superficial nature of the vessels, this has little impact on visualisation of the superficial vessels.

#### 3-D Imaging

A major limitation of US is that it is a 2-D assessment of a 3-D vessel/organ. Relying on operator interpretation 3-D images are normally reconstructed from multiple 2-D images using knowledge of orientation and position.^25^ Its main limitation is operator dependence on stability of 2-D images that are obtained. This may result in error of measurement or volume. The new 3-D Txs may overcome this limitation and provide the accuracy for true mathematical modelling. 3-D imaging is recognised in a vascular setting in the assessment of carotid plaque. This tool may be useful in the mathematical modelling of the anastomosis of the AVF. Volume analysis performed serially over the weeks subsequent to AVF formation may give an insight to the dynamic modelling process and allow the prediction of the criteria associated with successful fistula formation.^96^

## DSA

DSA is based on the acquisition of digital fluoroscopic images combined with injection of contrast material and real-time subtraction of pre- and post-contrast images. The technique was first developed in 1927 by the Portuguese physician and neurologist Egas Moniz at the University of Lisbon who applied it to the nervous system. The procedure became safer and widespread after the introduction of the Seldinger technique in 1953.^74^

### Technique

In DSA a first image of the area of interest (“mask”) is acquired and used as a reference to digitally subtract the “background” structures or tissues from the images taken after the contrast injection. In this way the vessels filled with contrast will appear black on a grey background, improving the contrast resolution of the image. The subtraction technique is based on the assumption that the tissues surrounding the vessels, apart from artifacts, do not change in position or density during exposure.^54^

#### Cannulation Approach

A rational approach to DSA depends on individual circumstances in each patient and facilities in each center. One critical element to obtaining good quality images of fistulas is the cannulation approach used. Imaging of the fistula can be obtained by injecting contrast media through the arterial or venous side of the fistula.

##### Venous Puncture

The direction of the puncture should be chosen according to presenting symptoms and physical/US exam of the patient: retrograde if the stenosis is supposed to be perianastomotic, and antegrade if the stenosis site is likely on the venous site. This is to avoid puncturing in the wrong direction in case an endovascular treatment is needed. The angiography of the inflow segment is then performed by injecting radiocontrast with simultaneous occlusion of the outflow segment by manual compression of inflating a blood pressure cuff. The contrast is allowed to flow retrograde into the inflow segment against the arterial pressure. The outflow segment can be imaged by releasing the manual occlusion of the same segment. Unfortunately, this technique is not entirely safe because of the risk of vascular rupture. The increased pressure on the walls of the fistula imposed by the occlusion of the outflow and forceful injection of contrast material can be detrimental and cause vascular rupture, especially immediately following an angioplasty or in non-maturing fistulas.^71^

A recent study by Chan *et al*. indicated the back pressure transmitted by the occlusion of the outflow segment, together with the force of the contrast injection, is responsible for a stretch effect with consequent dilatation of the segment.^21^ This all happens in a non-physiologic state, where flow and steal cannot be accurately assessed and results in an approximately 20% of underestimation of the inflow lesions.^19^, ^21^, ^51^ Another aspect to be considered is that the back pressure can open up collateral veins in the juxta-anastomotic region that may not be haemodynamically significant but create confusion for the treatment options.^51^ Finally, the venipuncture may induce spasm that is difficult to distinguish from stenosis.

##### Arterial Puncture

The retrograde puncture of the brachial artery can be used to bypass all the limitations of a venous retrograde occlusive angiogram and to visualize the inflow under physiologic condition. If treatment is needed, a second access from the venous side, should be performed. In this case the brachial artery access could be useful during the procedure providing a clear road map to perform therapeutic interventions, and may also be used for administration of glyceryl trinitrate and heparin as needed in the procedure. Unfortunately, the complications related to direct brachial artery puncture such as hematomas, nerve damage, distal ischemia, brachial artery thrombosis, and pseudoaneurysm formation make this approach prohibitive in haemodialysis patients.^92^

### Primary Uses in VA

DSA can be used not only for diagnostic, but also for therapeutic purpose, sometimes employing the same access used for the diagnostic exam. Transluminal angioplasty or endovascular stent positioning are a safer alternative to surgery with a higher chance of preserving the AVF, but still with an unimpressive long-term patency rates.^11^, ^13^, ^24^, ^35^, ^55^, ^60^, ^66^ DSA offers a high performance in evaluating the severity of arterial stenoses but it is invasive and has a high risk of complication.

#### Preoperative Assessment

**Figure 2:**
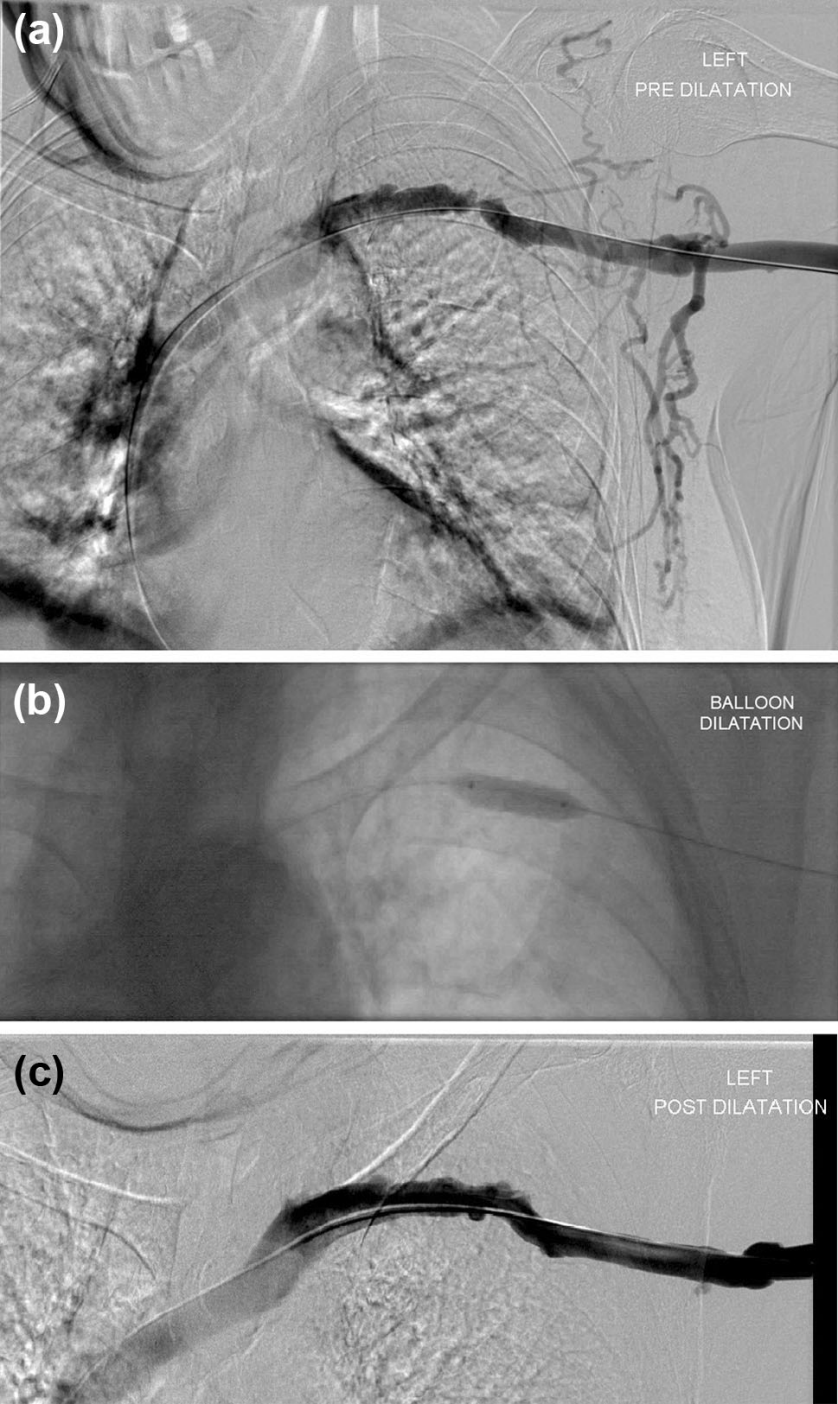
(a) Left arm angiogram shows a tight stenosis of the left subclavian vein, with marked visibility of the collateral branches. (b) A 10 mm standard balloon was used for the dilatation which (c) gave a good post dilatation result.

Guidelines suggest the use of MRI (to avoid the contrast media injection) or DSA in cases where the central veins have been previously cannulated to exclude the presence of constructing access ipsilateral to a central vein occlusion or significant stenosis.^48^, ^87^ DSA should instead be preferred to non-invasive diagnostic imaging studies in patients who present with acute symptoms of central venous stenosis and occlusion^6^; however, it is often through US assessment that initially indicates central venous stenosis and occlusion. An example of a stenosis of the left subclavian vein, and how it was treated with a 10 mm balloon, is shown in Fig. 2.

#### Post-operative Assessment

**Figure 3:**
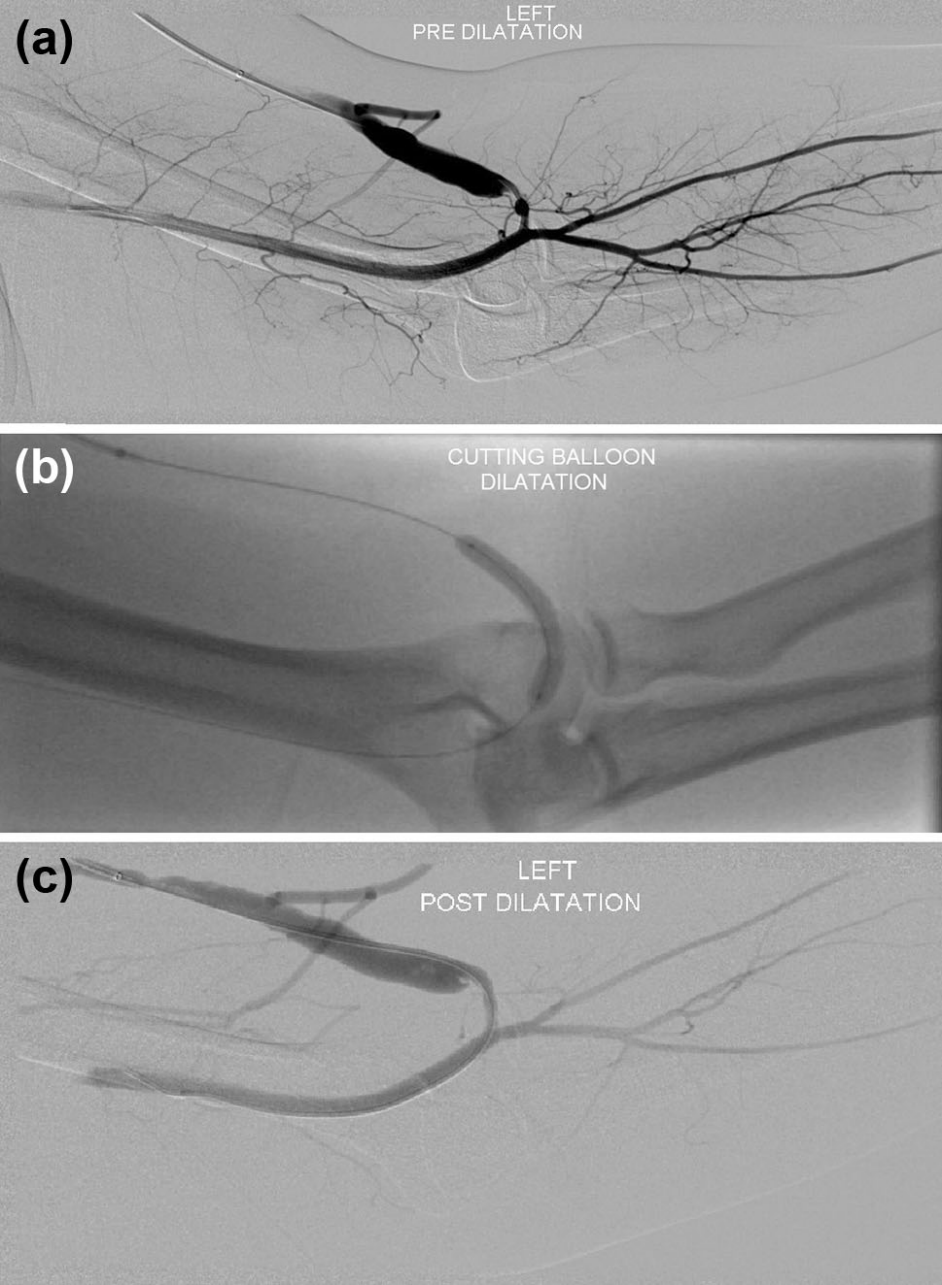
(a) Stenosis of a left brachiocephalic fistula, just central to the anastomosis. (b) A 6 mm cutting balloon was deployed at the stenosis site. (c) Good post dilatation results were observed.

A fistulogram may be useful to study non-maturing fistula at least after six weeks from the creation, whenever the AVF diameter is <6 mm or with a fistula blood flow of <600 mL/min.^43^, ^48^ DSA may be useful also in failing AVF when there is inflow or outflow stenosis(es) >50%. Figure 3 shows an example of a stenosis of a left brachiocephalic fistula and its treatment using a 6 mm cutting balloon. Aside from these situations, DSA can be used whenever an endovascular treatment is suspected to be necessary (presence of stenosis, “competing” veins, clotting, recirculation).^43^ DSA remains the gold standard for identifying the site(s) of venous obstruction and to have an overall assessment of venous thrombus.^6^

### Advantages/Disadvantages

DSA is the gold standard for assessment of the venous system. Prior to the AVF formation, venography is able to identify clinically occult veins which may be usable for AVF formation.^39^, ^63^ It can identify central vein stenosis and has been investigated as a potential adjunct to preoperative assessment of patients with ESRD referred for AVF formation. DSA is a dynamic exam and although it requires the use of contrast agent it is not any more invasive than cannulation during a dialysis session. If needed, DSA can be followed immediately by a percutaneous intervention, and thus is not only diagnostic but also suitable for treatment. Interventional radiology is preferred to surgery for the treatment of most of the cases of vascular access dysfunction thanks to its minimal invasiveness, better preservation of the patient’s venous reserve, and better outcomes for selected indications such as thrombosed autogenous fistulas. There is, however, a learning curve and not all hospitals are provided with a renal unit.

DSA is not without its limitations, chiefly, its use of ionizing radiation. Also, it would be relatively expensive and therefore, less readily available as US, since small hospitals may not be equipped with an angiography system or specialized staff trained for this procedure. While the angiogram acquisition lasts only a matter of seconds, the preparation of the patient can take several minutes (positioning of the patient, cleaning of the skin, cannulation) with an overall estimated time of 15–30 min per procedure. Because it is invasive, DSA needs to be performed in aseptic technique to avoid the risk of infection. The use of intravascular contrast is directly associated with nausea, vomiting, flushing and hypotension; these symptoms appear to be related to the osmolality of the agent used as they are seen less commonly in patients receiving the lower osmolality agents.^70^ Anaphylaxis is an unusual but more serious complication. It also appears to occur less commonly with the non-ionic contrast agents.^20^ The patient needs to be dialysed after the procedure to prevent toxicity of the contrast to the remaining functional nephrons and to non-renal tissues, even if the use of “low osmolar” contrast agents may not necessitate imminent dialysis.^70^ Contrast medium can be used in patients who are being dialysed but is relatively contraindicated in pre-dialysis patients as contrast nephropathy may precipitate acute renal failure. A valid alternative to avoid the risk of renal damage from contrast media in patients with advanced chronic kidney disease and ESRD is the use of carbon dioxide as contrast media.^37^, ^48^

### Future Directions

DSA was born as a diagnostic procedure but with the advent of interventional radiology and with the improvement of other diagnostic imaging techniques like US, CT and MRI, its use as a merely diagnostic tool is losing importance. Currently, DSA of an AVF is generally performed when a therapeutic intervention seems inevitable; whereas it should be performed solely for diagnostic purposes only in selected cases.

## MRI

For VA imaging, MRI is generally only used in the research setting because of its high cost. Successful MRI of AV access requires a number of features. First, the images need to be high-resolution in order to visualise the small vessels associated with the fistula site. Second, the contrast between the vasculature and the immediate surrounding tissues needs to be maximised, and a good signal-to-noise ratio is required in order to visualise the relevant structures with sufficient clarity. Finally, the images should be acquired relatively quickly in order to minimise artefacts associated with patient motion. The successful inclusion of these features requires the need for a number of ‘trade-offs’ associated within the imaging process.

The process of proton MRI is based on the selective excitation of magnetic dipoles of hydrogen atoms within biological tissue. The excited nucleus reradiates a radiofrequency (RF) signal, which is detected and recorded with radiofrequency coils that are tuned to the nuclear precessional frequency, i.e., the Larmor frequency. Spatially selective RF excitation is achieved by means of an RF pulse in the presence of static magnetic field gradients, which generates precessional frequencies that vary linearly with position within the gradient field. A radiofrequency transmitter pulse (delivered *via* an RF coil around the anatomy under investigation) is responsible for the nuclear excitation. The signal intensity fluctuation detected is a consequence of nuclear density along with the characteristic longitudinal and transverse time constants of tissue relaxation, known as T1 and T2 respectively. Images are generated by sequentially changing the strength of the gradient magnetic field and reading the time-varying free induction decay signal. The individual frequency components of this complex signal can be extracted by using Fourier Transform mathematics to form the final MR image.

### Technique

There are many different vascular techniques within MRI and some of them are described below.

#### Time-of-Flight (TOF)

**Figure 4:**
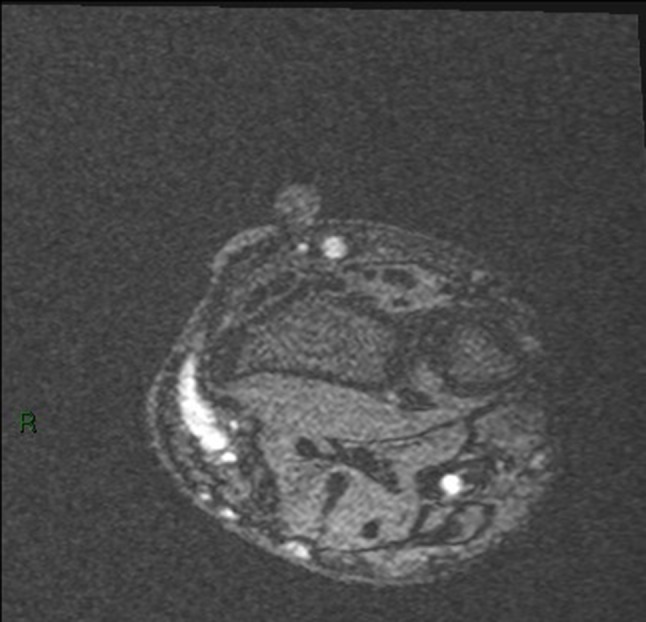
Typical TOF image showing a radiocephalic fistula swing segment as a bright region on the left. The round bright region adjacent to the arm at the top of the image is an oil capsule used as a reference marker.

**Figure 5:**
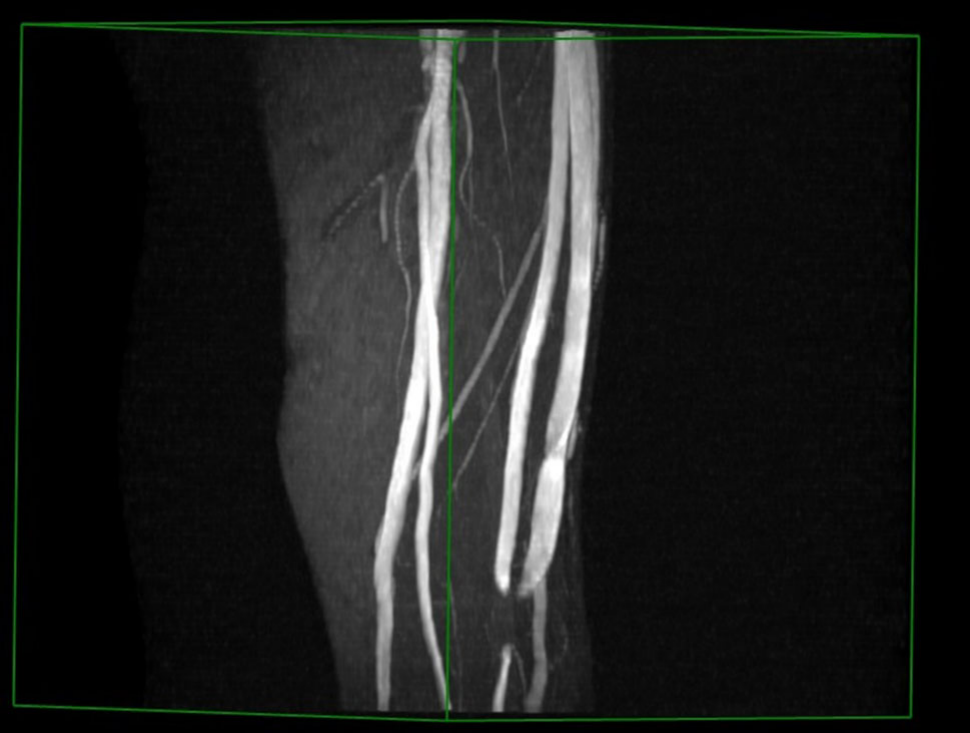
A snapshot of a 3-D reconstruction of the vasculature from TOF images at the elbow region of a patient’s brachiocephalic fistula. Note the signal dropout at the anastomosis because of flow turbulence.

The TOF sequence is a Gradient Echo (GRE) sequence with a very short repetition time (TR) relative to the T1 of stationary tissues and is often referred to as “bright blood imaging”.^50^ A typical example of a TOF image showing a radiocephalic fistula swing segment is shown in Fig. 4. Also, a separate 3-D reconstruction from TOF images of a patient’s brachiocephalic fistula is shown in Fig. 5. The TOF effect is only possible when the plane of the imaging slice is perpendicular to the direction of the blood flow. Figure 5 shows some signal drop out which can occur at the anastomosis site because of turbulent blood flow which is not perpendicular to the plane of the imaging slice. This is an important limitation in VA imaging since the flow within a fistula does not stay within one direction as it is turned through the swing segment.

#### Contrast Enhanced MR Angiography (CE-MRA)

CE-MRA techniques involve the use of a contrast medium (usually gadolinium-based), which is intravenously infused as a bolus in order to reduce the T1 of the blood. The reduction in T1 of the blood translates into a hyperintensity of flow signal over other surrounding tissue. The success of an MRA with contrast bolus depends on the synchronization between acquisition of data and concentration of the contrast medium in the vascular district of interest.^50^

#### 2-D Phase Contrast (PC-MRI)

**Figure 6:**
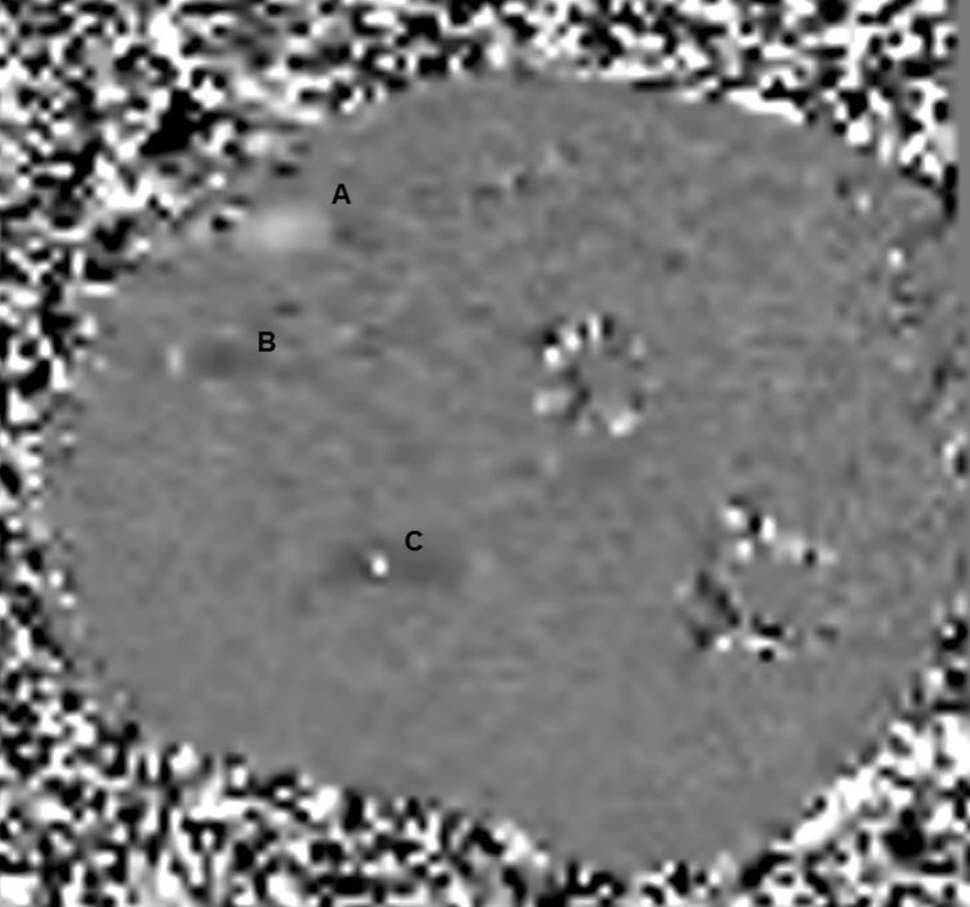
A snapshot of a VENC showing the radial artery as the light grey region (a) and the vein as a dark grey region (b), and some aliasing in the ulnar artery (c) because the velocities are outside the VENC range.

The PC-MRI method uses the phenomenon of MR phase shift as the source of the contrast and the images produced are sensitive to flow. In phase contrast imaging, a positive flow-encoding gradient is applied first, and this is followed by an equal and opposite negative one. Stationary proton spins which retain the same phase information will not be affected by the application of this positive/negative gradient pair, but moving proton spins will exhibit a net dephasing effect that is related to the strength of the applied gradients. The strength of the applied gradients can be controlled by the process of velocity-encoding (VENC) where the chosen VENC is able to assign the fastest flow with a nominal phase shift of 180°. PC-MRI is used with these VENC techniques to determine the flow velocity of the blood.^50^ Figure 6 illustrates an example of VENC.

#### Multi-Echo Data Image Combination (MEDIC)

The MEDIC method is a high spatial resolution 3-D gradient echo sequence which conveys T2* (effective T2) weighting to the resulting image. This sequence is useful in some cases for vascular imaging since the TR is long which enables the in-flow effect to contribute towards hyper-intense blood signal. It is possible to acquire these images in 3-D at high resolution (typically 0.5 mm in-plane) and with a very small slice thickness in order to highlight the vessels clearly. Typical scan times using this sequence are about 5–6 min though, so it is important to ensure that the patient is comfortable and instructed about the importance of remaining still during the acquisition.

#### Black-Blood MRI

Black-blood MRI, so known as the signal from blood is supressed rather than enhanced rendering it black in the resulting images, has an added benefit of providing information not only on the vessel morphology but also on wall thickness. It can produce a relatively high resolution ~300 μm in plane and a slice thickness of 1.5–2 mm.^78^ Using this technique stationary fluids and fatty structures within the slice as appear as hyperintense, while the blood signal remains hypointense.

### Primary Uses in VA

MRI can be used without contrast and can gives excellent anatomical detail of veins within the arm. These advantages mean that magnetic resonance venography (MRV) can be used where there is a suspicion of central vein stenosis and in patients with multiple previous access attempts.^18^ Additionally, some predialysis patients may have minimal renal function leaving them at risk of iodinated contrast causing acute renal failure. In these cases MRV may be more suitable instead of using CT or DSA, however, its use is limited for AV access assessment because of accessibility and expensive examination cost.^53^ CE-MRA can identify the lumen of upper extremity vessels and be more accurate in diameter determination in comparison with DUS.^65^

The use of non-contrast MRA for VA imaging tends to be restricted to the research environment because MRI is considerably more expensive than other imaging methods, such as US. US is also more portable and accessible. Also, to acquire the best image it is recommended that a patient’s arm should be positioned in the isocentre of the MRI bore, the practicalities of which, especially with frail patients, are not trivial. However, on the positive side, the contrast, signal-to-noise and resolution associated with MRI can result in excellent images of the VA site when required. The two ‘standard’ approaches for patient positioning are: (i) in the prone position, with their arm above their head (the ‘superman’ position), and (ii) in the supine position with their arm down by their side. The former method is susceptible to the patient suffering from temporary blood loss from within the veins (‘pins and needles’ phenomenon) and with the latter method it is difficult to position the arm centrally within the homogeneous region of the magnetic field. In preliminary work that has been completed looking with the same set of healthy volunteers in both of these imaging positions it has been established that the supine position is preferable, since it is the more patient friendly and does not cause the same ‘blood loss’ phenomenon within the venous structures.

Extensive use of MRI for the generation of geometries for computational modelling and image-based computational fluid dynamics (CFD) can be found in biomedical engineering area, and the generation of geometries from MR images is well established.^7^, ^8^, ^28^ A brief overview of the progress achieved in the field of image-based CFD studies was done by Steinman and Taylor^79^, ^85^ with applications in research assessing WSS-based metrics.^78^

### Advantages/Disadvantages

MRI has several key advantages for VA imaging: it’s non-invasive, does not require the use of ionising radiation, provides excellent tissue contrast and resolution, and can be undertaken with or without contrast agents. The latter advantage is important as contrast agent use should be restricted in patients with impaired renal function. Unlike US, MRI is not limited by bone structures and air pockets within the body, and can be used to image the central venous system. A comparison study between MRI and US from Glor *et al*. concluded that black blood MRI and 3-D US are interchangeable for carotid flow reconstructions.^29^ In a similar study, Goubergrits *et al*. compared MRI and CT using a silicon model of the left coronary artery main bifurcation. The calculated average WSS shows high correlation and agreement among the modalities.^31^

CE-MRA can be used, as described above, for detailed analysis of upper extremity vessels; however, whilst is can provide superior anatomical detail and excellent multi-planar imaging two disadvantages are its susceptibility to artefacts (e.g., stents, some AVG), and also concerns around the development of nephrogenic systemic fibrosis secondary to gadolinium contrast administration. More generally, the main disadvantage of MRI is its cost which also, consequently, makes it less accessible in comparison with US. Another disadvantage of MRI is the time-consuming nature of the modality in acquiring adequate diagnostic images and therefore, resource implications. In contrast, US, with an experienced sonographer, can produce accurate diagnostic images in real time. Additionally, the bore size of MRI machines can limit the size of patient that can be accommodated comfortably for assessment which is an issue for obese patients. This also impacts patients that may find it difficult to remain in the scanner for the considerable time it takes to acquire adequate images, especially if they suffer from claustrophobia. All of these disadvantages generally limit MRI’s use in VA to the realm of research where the high resolution and accuracy are required.

### Future Directions

Although MRI is a powerful imaging technique, in terms of diagnostic and surveillance for VA, its usage is limited until now; however, there are several promising areas of research which hold great potential for the assessment of VA. Recent technological advances in non-contrast vascular MR include the development of 4D phase contrast for imaging blood flow and velocities over a 3D field of view. This technique is often applied to the aortic arch^81^ to assist with flow modelling, and also more recently to the cerebral veins.^73^ Recent advances in 4D flow have included the development of multiple velocity encoding 4D acquisitions allowing detailed and accurate assessment of both high and low velocity structures opening up this technique to the possibility of both fistula and central venous assessment.^34^ However, further refinement is likely to be required for VA studies – including the need for improvements in the spatial resolution and faster imaging times.

In addition to the above, non-contrast MR methods that rely on the in-flow of blood during the systolic phase of the cardiac cycle are also anticipated to form the basis of future developments as the technology enables these images to be acquired faster and at a higher resolution. Currently the main use for these techniques is for arterial imaging (with associated saturation of the venous flow),^99^ but it should, in theory, be possible to retain the venous information by dispensing with the venous saturation or acquiring interleaved images (with venous flow present and absent) that could then be subtracted from one another. Commercial examples of these newer techniques include Time-SLIP (Time Spatial Labelling Inversion Pulse), NATIVE (Non-contrast MRA of Arteries and Veins), QISS (Quiescent Interval Steady State), TRANCE (Triggered Angiography Non-Contrast Enhanced) and Inhance Inflow Inversion Recovery (IFIR).

The use of gadolinium-based contrast agents is anticipated to continue in those patients who have sufficient renal function (to mitigate risk associated with nephrogenic systemic fibrosis).^42^ The further development of commercial time-resolved MRA techniques, such as TRICKS (Time Resolved Imaging of Contrast Kinetics), TWIST (Time Resolved Angiography with Stochastic Trajectories) and 4D TRAK (4D Time Resolved Angiography using Keyhole), in combination with faster parallel imaging in two dimensions is anticipated to enable repeated scanning of 3D datasets to be completed within sub-second temporal resolutions. Additionally, the contrast agent Ferumoxytol is an ultra-small superparamagnetic iron-oxide (USPIO) nanoparticle which has been used in the chronic kidney disease population since 2009 as a treatment for iron deficiency anaemia. It has recently become of great interest due to its paramagnetic effects and its biodistribution, with the compound being distributed almost exclusively within the blood pool where it has a half-life of 15 h.^26^ This means it can be used for simultaneous arterial and venous imaging, and the stable vascular properties mean that acquisition no longer has to be confined to the typical 20 s arterial phase window of currently used gadolinium based contrast agents, allowing for higher resolution image acquisition. This has successfully used in a small number of both adult and paediatric patients with ESRD for vascular access and renal transplant assessment with promising early results.^57^, ^75^

Finally, the development of higher field 7T MRI scanners is anticipated to offer potential for improved VA imaging. The higher field strength should enable better signal-to-noise and spatial resolutions to be achieved, and even allow for clearer visualisation of the vessel wall structures.^47^ Conventional techniques such as TOF MRA may also be better at 7T since the T1 relaxation times are longer at the higher field which results in clearer visualisation of the vasculature in-flow due to the improved background suppression of stationary tissues within the imaging slice. It is also possible to use high resolution T2* gradient echo contrast (Susceptibility Weighted Imaging—SWI) at 7T in order to depict venous vessel structures with exquisite detail.^17^ The basis of the contrast is achieved from susceptibility differences between the de-oxygenated venous blood and the surrounding soft tissues.

## CT

**Figure 7:**
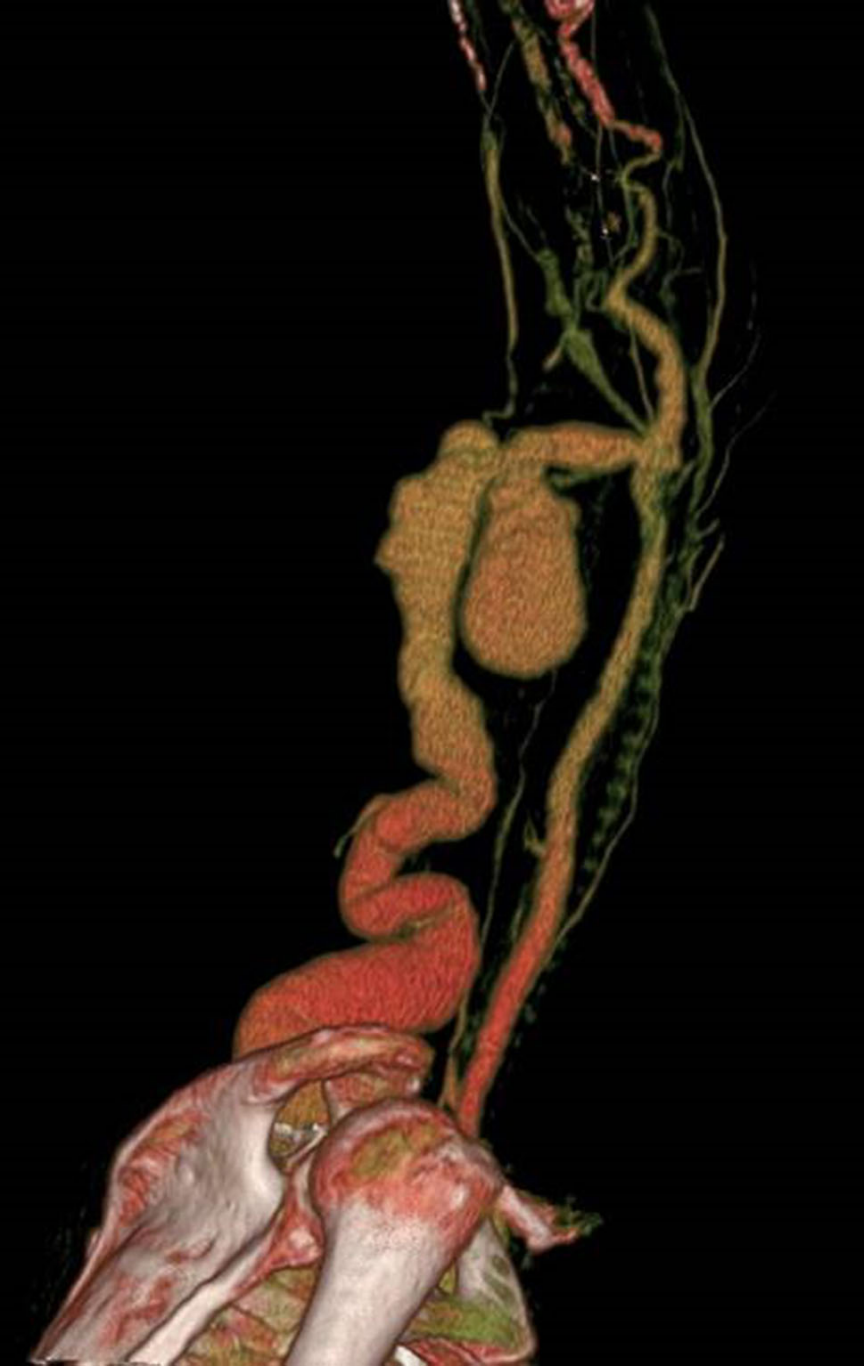
3D reconstruction CT scan of a left brachiocephalic AV Fistula. The patient presented with localized gross bruising and tenderness 24 h following dialysis. A pulsatile swelling was evident on clinical examination. Ultrasound examination was not possible due to patient discomfort. The image adequately demonstrates a large pseudoaneurysm arising from the proximal aspect of the AV fistula. This image provided vital pre-operative road mapping information to the surgeon before operating to repair this abnormality.

Modern Multi-detector CT (MDCT) scanners have extremely short acquisition times and high spatial resolution. This high spatial resolution has been shown to be particularly useful when considering CT angiography for the vascular assessment.^46^ This application is directly transferrable to the AV access setting and CT is ideally suited for imaging dysfunctional AV access and facilitating the decision making around future treatment.^1^, ^38^, ^46^, ^104^ 3-D reconstructions and Maximum Intensity Projection (MIP) images of CT data are extremely useful in case conferences especially if surgical intervention is required (Fig. 7). At some institutions the use of CT has proven useful when utilized as a problem-solving tool in complex cases where conventional imaging has not provided adequate information.

### Technique

Ideally the arm with the AV access should be fully abducted to avoid artefact from the body and reduce radiation dose, which would be increased if it were positioned by the patient’s side. Intravenous access should be obtained in the contralateral limb. Typically when standard CT angiography protocols are applied the contrast bolus is injected with a five second delay following tracking off the aortic arch. Depending on the clinical information the whole thorax may be covered in the scan to access the entirety of the central venous system. Thin collimation (typically 1 mm) facilitates high resolution multi-planar reformatting and 3-D reconstruction.

### Primary Uses in VA

#### Assessment of Stenosis

Several authors have demonstrated the feasibility for CT assessment of dysfunctional AVF and AVG with sensitivity and specificity values for detecting stenosis ranging from 90 to 99% and 93 to 98%, respectively.^38^, ^46^ These results are encouraging and are comparable with DSA.^38^, ^46^ If a stenosis is non-concentric it could be overlooked on DSA, which only provides 2-D information unless multiple oblique angiographic views are acquired which contribute further to cumulative radiation dose. In contrast, CT provides 3-D information about the whole vessel or access in a single acquisition.

**Figure 8:**
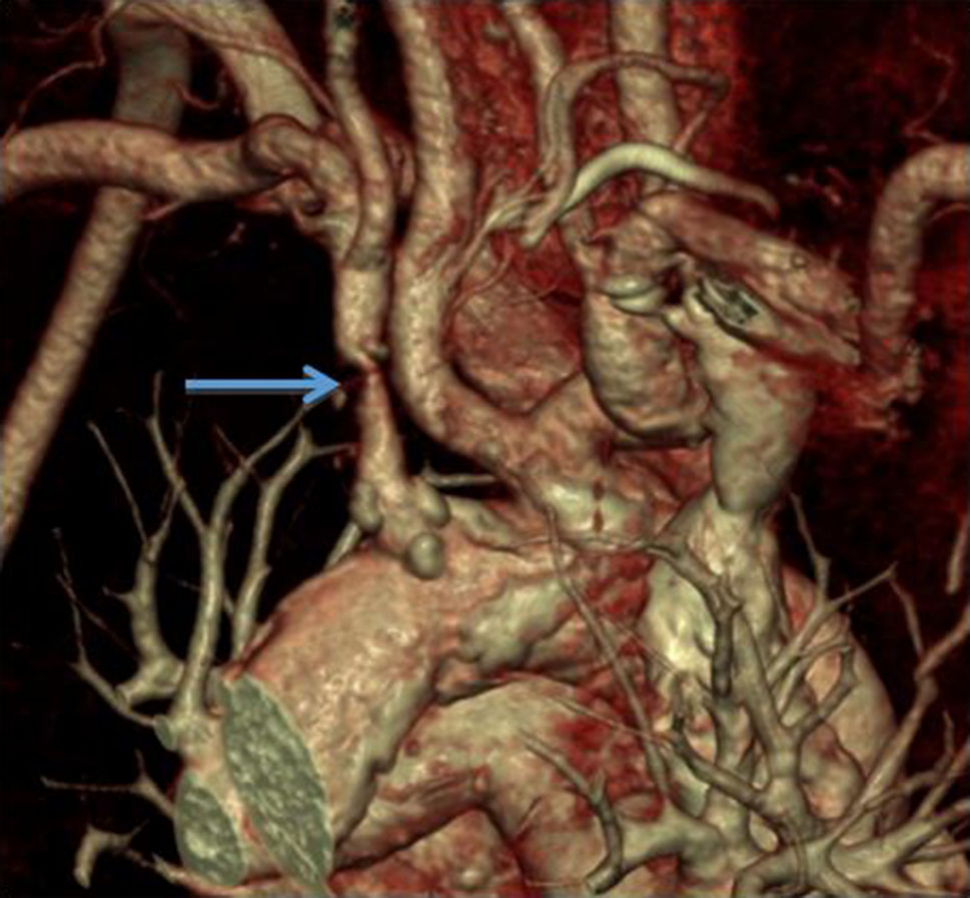
3D reconstruction CT scan of an aortic arch in a patient with a poorly maturing AV fistula. A proximal subclavian artery stenosis was identified (arrow) and subsequently treated with an endovascular stent.

Whilst MDCT cannot be utilized directly for therapy in vascular access, it can provide powerful and accurate planning information in certain circumstances before DSA and endovascular intervention are considered. One of main advantages of CT is the ability to visualize and assess the whole dialysis access circuit from the left ventricle to the right atrium. Proximal inflow stenosis is difficult to diagnose with Doppler US and standard DSA would not necessarily cover this region if focusing on the AVF or AVG itself. Proximal arterial stenosis can lead to poor maturation or arterial steal symptoms, and is often overlooked (Fig. 8).

#### Central Vein Assessment

CT has superiority in assessing the central veins and can accurately demonstrate extrinsic compression, which may be overlooked on DSA or difficult to assess with Doppler US (e.g., central vein compression from bony structures). The subclavian vein can be compressed at the costoclavicular junction, which is insignificant in the normal subject but can lead to morbidity in an AV access patient with an arterialized vein secondary to an AVF or AVG. Arm swelling and pain is a typical presentation in this group along with high venous pressures and inadequate dialysis. Left untreated this can lead to subsequent thrombosis and eventual loss of the access. Accurate diagnosis and distinction between true luminal stenosis and extrinsic compression is essential to guide further intervention, which may include rib resection in the latter.^40^ As a side note, in cases of central venous stenosis, be it luminal or extrinsic, evaluation of inflow is always important as the symptoms of central venous stenosis are often exacerbated by high inflow access which may be more appropriately managed with inflow reduction surgery such as banding.

#### Other Applications

**Figure 9:**
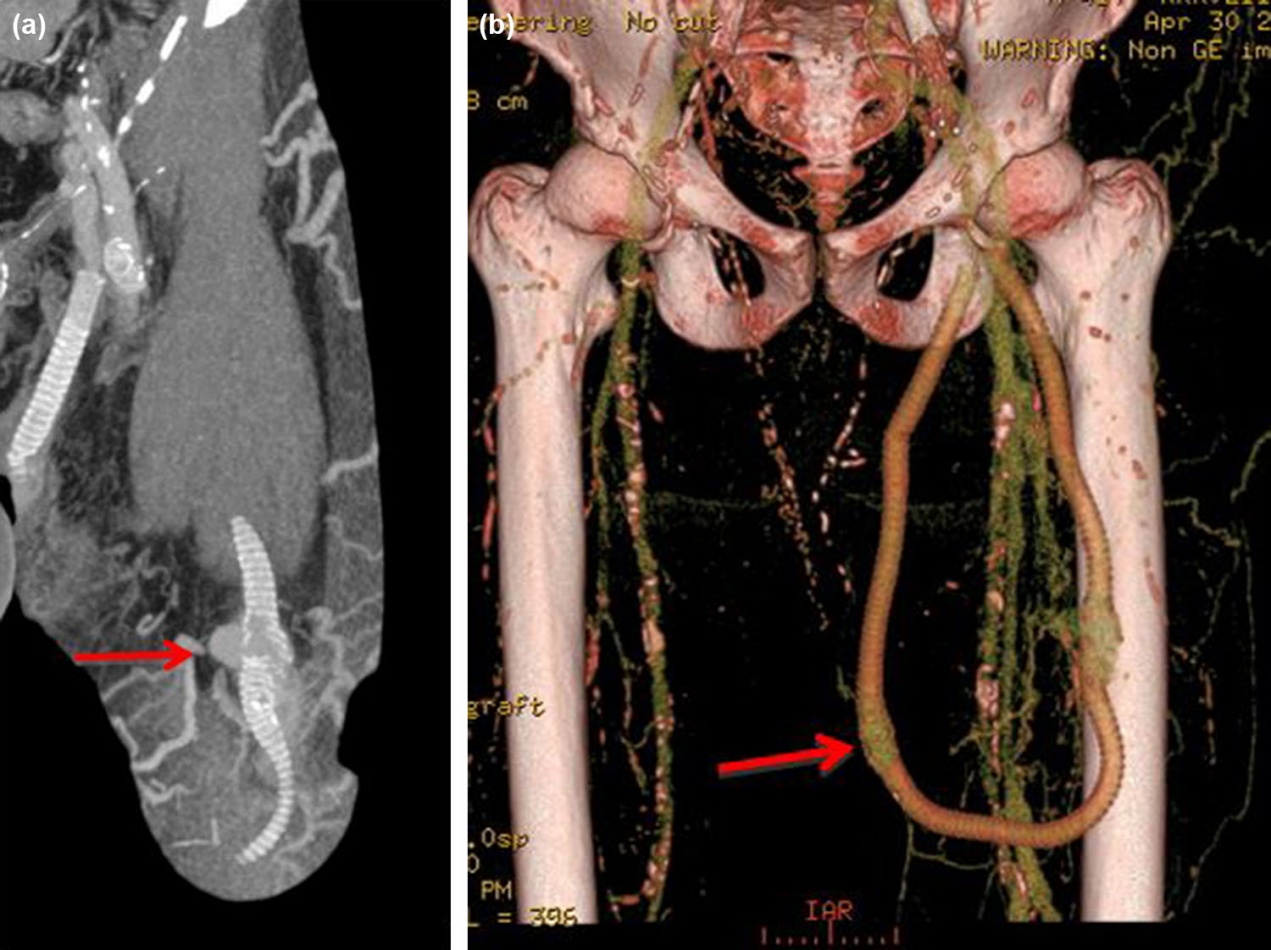
(a) Maximum intensity projection (MIP) image of a left loop thigh AVG in a patient who presented with spontaneous bleeding over the AVG. It demonstrates localized breakdown of the graft material and pseudoaneurysm formation (arrow) associated with repetitive needling at the same site. This was urgently treated with insertion of a stent-graft. (b) 3D reconstruction of the same patient also shows early AVG breakdown in the medial limb of the graft (arrow).

Pseudoaneurysms can cause AV access dysfunction. These are more common in AVG than AVF and usually develop as a result of repeated needling in the same position, leading to breakdown of the graft material and subsequent pseudoaneurysm formation which can predispose to infection or rupture if left untreated (Fig. 9).

Calcification is prevalent in the haemodialysis population and an important consideration when planning an AVF or AVG. CT can accurately assess the amount of calcification in the artery being considered and provide a useful road map to the surgeon in patients known to have a high vascular calcification burden. In this scenario intravenous contrast may not always be required and therefore, plain CT would be a safe application in the pre-dialysis patient being worked up for their first vascular access. Toussaint *et al*. concluded in a small retrospective review of 28 scans, that it may be possible to obtain accurate vascular calcification scores from CT fistulograms, which in turn could be used to determine cardiovascular risk assessment in dialysis patients.^91^

### Advantages/Disadvantages

CT produces images with high spatial resolution that are considered advantageous in both a clinical, in AV access assessment, and a research setting, generation of accurate 3-D geometries of vasculature. The main drawback of CT is the use of ionizing radiation but with the evolution of CT technology radiation doses are decreasing dramatically with newer algorithms still providing high spatial resolution images and superior diagnostic information compared to standard radiological examinations such as plain X-rays.^82^ CT assessment of dialysis access requires a bolus of iodinated intravenous contrast and AV Dialysis access is usually created some months in advance of the new access patient requiring dialysis. In these cases where the patient is not already requiring dialysis any residual renal function could be compromised further by the use of intravenous contrast and therefore, CT would be disadvantageous in this scenario.

### Future Directions

**Figure 10:**
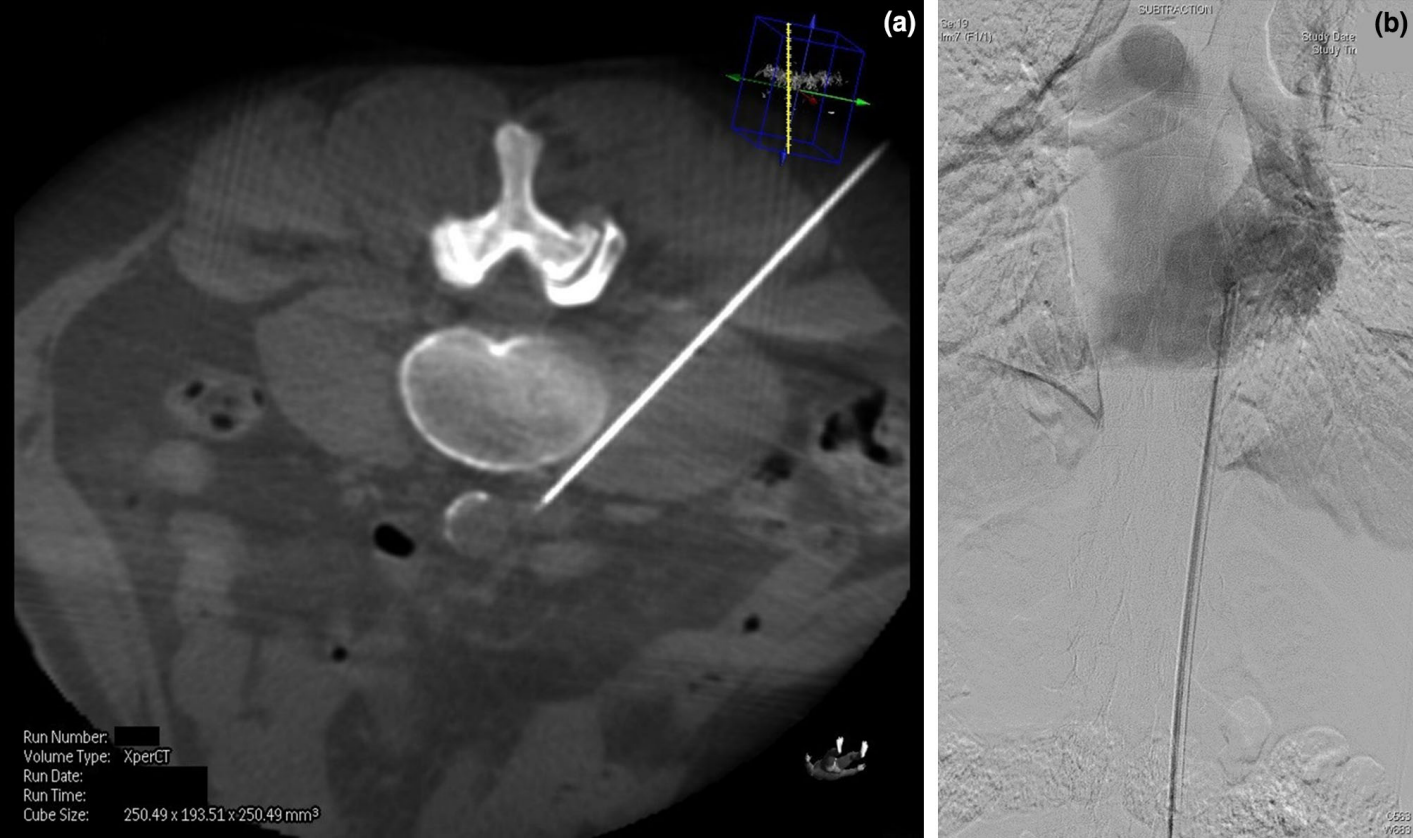
(a) Single cone-beam CT image taken during placement of a translumbar vena cava dialysis line in a patient with no further peripheral vascular access options. The image was used to safely and accurately puncture a stenotic inferior vena cava (IVC) *via* the tranlumbar route. The image shows the needle is the correct position with the tip in the IVC. (b) The tunneled catheter was subsequently placed using the Seldinger technique. The tip of the catheter can be seen adequately positioned within the right atrium on this single DSA image.

The diagnostic application of MDCT in the haemodialysis patient has been addressed here, but with evolving technology it is possible to obtain limited CT information (cone beamed CT) from modern angiography machines. This cross-sectional information can be acquired in real time during an interventional radiological procedure and aid the operator by providing additional 3-D information not available on the standard 2-D fluoroscopy of DSA. Cone-beam CT can provide a vascular road map in cases of complex vascular access intervention^86^ (Fig. 10).

## Conclusion

Dysfunctional haemodialysis access requires rapid and accurate assessment to avoid subsequent morbidity and mortality with non-invasive imaging modalities considered the standard in the first instance. US is the principal imaging modality used because it is cheap, accessible, non-invasive, and provides accurate information on the dynamics of the access vessel whilst not exposing the patient to ionising radiation; however, it is very operator dependent. CT can fulfil this role in certain circumstances and several published studies have shown a high degree of accuracy, although its main drawback is the inherent use of ionising radiation. With evolving MDCT technology the accuracy of diagnosis is likely to improve with a paralleled reduction in radiation dose, widening the application of CT in vascular access in the future. DSA is predominantly used when a VA site requires intervention and is the gold standard for assessment of the venous system, particularly the central vein which cannot be assessed by US. In recent years MRI and CT are being utilized to non-invasively assess failing AV access with good evidence emerging to support their development and application in this area.^1^, ^38^, ^44^ A safe and reliable haemodialysis VA has proved elusive. The unique haemodynamic environment created by an AVF or AVG coupled with the particular underlying medical issues associated with ESRD make improving VA outcomes challenging. Further investigation of the haemodynamic environment, and the biological processes that can result in VA failure is warranted. No one imaging modality reviewed here can provide the complete picture and it is anticipated that the future lies in a more integrated approach using information gleaned from the several modalities available.
